# Multimodal Treatment Including Intracavitary Chemotherapy for Recurrent Pediatric Pleuropulmonary Blastoma

**DOI:** 10.7759/cureus.20584

**Published:** 2021-12-21

**Authors:** Merieme Habti, Gervaise Hubert, Monia Marzouki, Nelson Piché

**Affiliations:** 1 Pediatric Surgery, Université de Montréal, Montreal, CAN; 2 Pediatric Oncology, Centre Hospitalier Universitaire Sainte-Justine, Montreal, CAN; 3 Pediatric Surgery, Centre Hospitalier Universitaire Sainte-Justine, Montreal, CAN

**Keywords:** pleuropulmonary blastoma, intracavitary chemotherapy, pleuropulmonary blastoma recurrence, intrapleural cisplatin, target therapy oncology

## Abstract

Pleuropulmonary blastoma (PPB) is a rare pediatric cancer, and although there have been improvements in its treatment approach, recurrences retain a very poor prognosis. Here, we report a case of a 30-month-old female who survived recurrent PPB after undergoing surgery, adjuvant chemotherapy, intrapleural cisplatin infusion, and targeted therapy through whole exome sequencing (WES). Intrapleural cisplatin infusion and target therapy appear to be safe and can be considered in a multimodal approach for the management of recurrent PPB.

## Introduction

Although rare, pleuropulmonary blastomas (PPBs) remain the most common primary pediatric pulmonary malignancy, with a relatively poor prognosis [1]. Similar to most solid pediatric cancers, PPB recurrence is associated with a grim prognosis [2]. Herein, we report a case of recurrent PPB successfully treated with surgery, intrapleural cisplatin infusion, adjuvant systemic chemotherapy and radiotherapy, and targeted therapy. This case underlines the importance of a multimodal approach for recurrent PPB and the potential role of novel treatment techniques in managing this challenging malignancy.

## Case presentation

**Figure 1:**
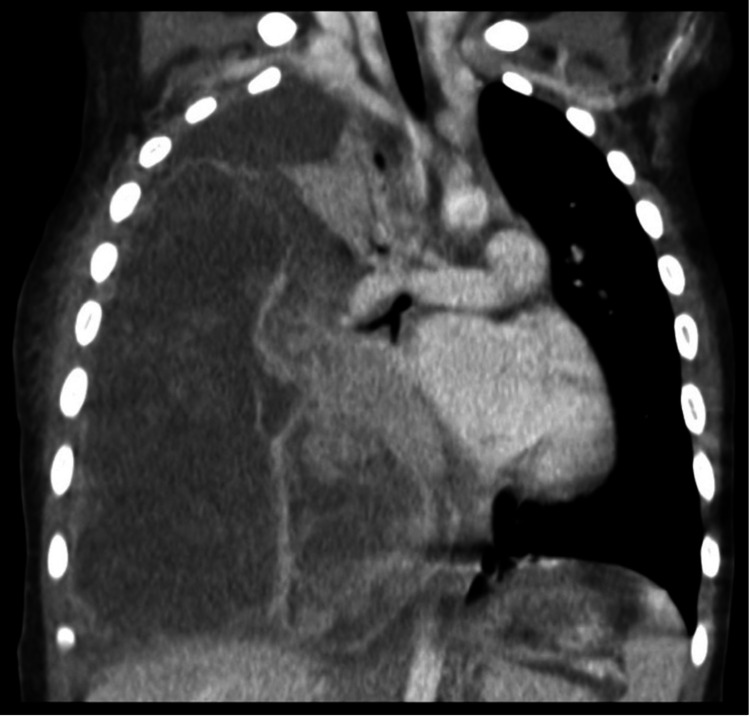
Chest CT at diagnosis

A 30-month-old female presented emergently with respiratory distress, desaturation, and fever. Past medical and familial histories were negative. Chest CT demonstrated a moderate right pleural effusion, left mediastinal shift, and a voluminous multinodular mass measuring 13 × 10 × 10 cm with cystic areas in the right pleural space (Figure 1).

The patient was admitted, and a right thoracotomy was performed. A malignant-appearing friable tumor with a large pleural effusion was identified. Resection of the mass was performed by wedge resection of the right lower lobe. Histopathologic analysis established the diagnosis of a type II PPB. Extension workup revealed no brain metastases, no bone marrow involvement, and no other suspicious capitation sites on the PET scan. The postoperative course was favorable, and chemotherapy using the ifosfamide, vincristine, actinomycin D, and doxorubicin (IVADo) protocol was initiated according to the suggested protocol of the International Pleuropulmonary Blastoma Registry [3]. Genetic counseling was obtained, and testing for germinal DICER1 mutation proved to be positive about eight months after initial diagnosis. Since then, the patient underwent usual surveillance without identification of DICER1 germline mutation-associated new tumors [4]. The child completed a year of chemotherapy for a total of 12 cycles with surveillance CT scans every three months for the first year and every six months for the second year.

**Figure 2:**
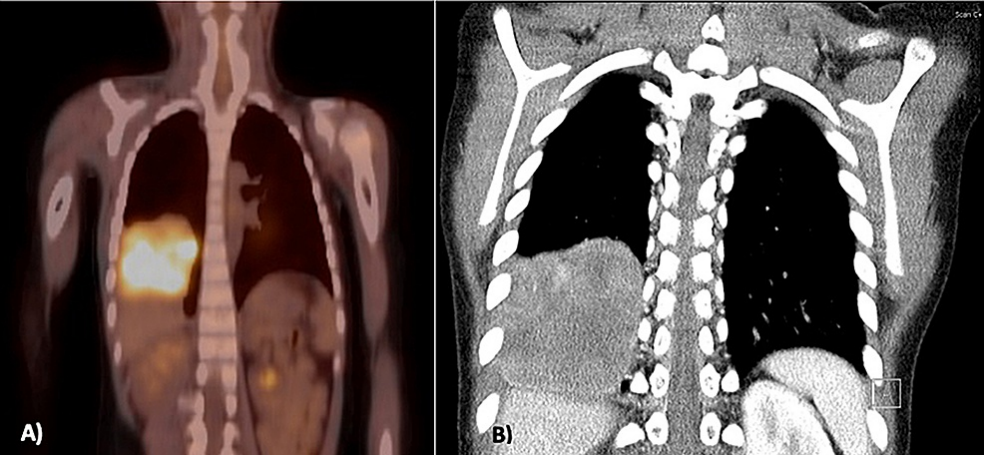
Imaging at the first relapse A) PET scan, B) CT scan

She relapsed locally at about six years of age when her PET and CT scans revealed a suspicious 69 × 39 × 65 mm mass in the right pleural cavity (Figure 2). After multidisciplinary discussion and with consent from the parents, a second thoracotomy was performed, and the lesion was resected en bloc by a right lower lobectomy.

This was followed within three hours of surgery by intrapleural cisplatin infusion. Immediately after surgery, aggressive IV hydration was begun using D5% 0.45 saline at 125 mL/m^2^/hour. Once the patient was deemed stable, 75 mg/m^2^ of cisplatin diluted in warm normal saline to achieve a total volume of 180 mL was administered intrapleuraly via the chest tube. The solution was given over 15 minutes and was left in the intrapleural cavity for four hours. During this time, patient positioning was changed every 15-30 minutes to improve cisplatin distribution in the pleural cavity. After four hours, the liquid was drained through the chest tube. Postoperative evolution was uneventful. The chest tube was ceased on postoperative day five, and the patient was discharged on postoperative day nine.

Adjuvant chemotherapy using vincristine, actinomycin, and cyclophosphamide (VAC) was begun one week postoperatively and administered for a total of 10 cycles. She also received local radiotherapy, with 25 fractions of 1.8 Gy (total: 45 Gy). She was also enrolled in the TRICEPS study, a prospective study aiming to use molecular profiling to better target treatments for hard-to-treat pediatric cancers [2]. Whole exome sequencing (WES) of her tumor revealed very high expression of fibroblast growth factor receptor 1 (FGFR1) and catenin beta 1 (CTNNB1) genes. Celecoxib (100 mg DIE) was added to her treatment to target the CTNNB1 mutation.

**Figure 3:**
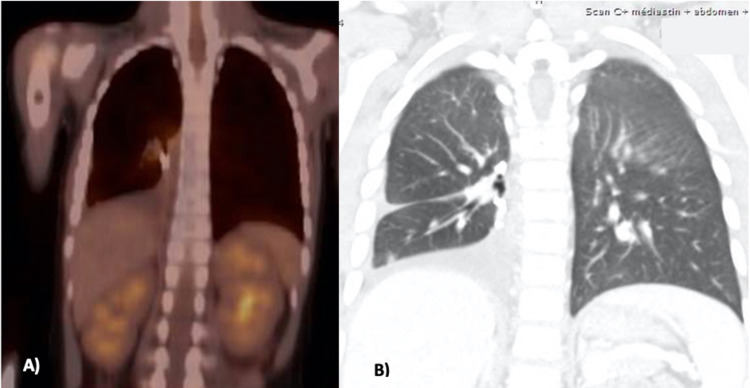
Imaging at the second relapse A) PET scan, B) CT scan

Seven months later, on posttreatment imaging, CT showed persistent linear opacities in the right lower lung, and PET also revealed persistently increased metabolism adjacent to the surgical staple line (Figure 3). These findings were compatible with postoperative changes, but a second recurrence could not be excluded, and consequently, sorafenib (100 mg BID) was added to target the FGFR1 gene amplification.

Celecoxib was stopped two years after introduction at the parent’s request, although no major side effects were noted. As with sorafenib, she temporarily showed slightly elevated liver enzymes and minor gastrointestinal symptoms (e.g., diarrhea and constipation), but the treatment has been well tolerated overall and is still ongoing. The patient is now 10 years old, and all follow-up imaging shows no relapse of her PPB almost four years after resection and intrapleural cisplatin infusion.

## Discussion

PPB is a rare pediatric tumor usually arising from the lung or pleura. The earliest stage of PPB is a lung cyst, and PPBs can evolve into three types: type I (purely cystic), type II (cystic/solid), and type III (solid) [1]. This lung malignancy is known to be associated with DICER1 syndrome in about 66% of cases, which is linked to other pathologies such as ovarian tumors, multinodular goiter, or thyroid carcinomas [5].

Treatment varies depending on the PPB type but mainly relies on a combination of surgical resection, adjuvant chemotherapy, and/or radiotherapy [6].

Treatment options for recurrent cases are not firmly established and should be tailored individually for each patient. Our case report shows the potential benefit of a multimodal approach using both the conventional approach and more novel methods such as intracavitary chemotherapy and molecular profiling.

Intracavitary cisplatin perfusion, both intrathoracic and intraperitoneal, has been studied mainly in the adult population to improve local control, with fewer long-term sequelae than systemic chemotherapy [7-9]. This treatment method has been widely described for adult malignant mesothelioma, demonstrating both improved survival and reduced systemic toxicity [10]. The literature on intracavitary cisplatin use in pediatric patients is limited, but a potential benefit has been shown in managing rare pediatric tumors when unresectable or recurrent disease is contained within a body cavity [11,12]. In one study, two of three long-term survivors were treated with intracavitary cisplatin at diagnosis [11]. We chose to perform early postoperative intrapleural cisplatin perfusion to ensure patient hemodynamic and respiratory stability prior to intracavitary perfusion and optimize cisplatin distribution in the pleural space before adhesion formation. We opted for a closed technique, as opposed to an open intraoperative approach, to decrease the risk of cisplatin spillage.

## Conclusions

Intrapleural cisplatin infusion appears safe and is a well-tolerated treatment option in children. It could be considered in a multimodal treatment plan to address recurrent PPB in the hopes of attaining long-term survival, but obviously, more studies are needed to better characterize this novel approach. Targeted therapy through whole exome sequencing also seems to be a promising novel approach for hard-to-treat pediatric cancers.
